# Cost-effectiveness analysis of CTZ/TAZ for the treatment of ventilated hospital-acquired bacterial pneumonia and ventilator-associated bacterial pneumonia in Japan

**DOI:** 10.1186/s12913-024-10883-7

**Published:** 2024-03-28

**Authors:** Risako Takaya, Nobuyoshi Mori, Eiko Saito, Sachiko Ohde

**Affiliations:** 1 Graduate School of Public Health, St. Luke’s International University, 10-1 Akashi-Cho, Chuo-City, Tokyo, 104-0044 Japan; 2 St. Luke’s International Hospital, Tokyo, Japan; 3 National Center for Global Health and Medicine, Tokyo, Japan

**Keywords:** Ceftolozane/tazobactam, Meropenem, Ventilated-hospital-acquired bacterial pneumonia/ventilator-associated bacterial pneumonia, Cost-effectiveness analysis

## Abstract

**Background:**

Resistant bacterial infections, particularly those caused by gram-negative pathogens, are associated with high mortality and economic burdens. Ceftolozane/tazobactam demonstrated efficacy comparable to meropenem in patients with ventilated hospital-acquired bacterial pneumonia in the ASPECT-NP study. One cost-effectiveness analysis in the United States revealed that ceftolozane/tazobactam was cost effective, but no Japanese studies have been conducted. Therefore, the objective of this study was to assess the cost-effectiveness of ceftolozane/tazobactam compared to meropenem for patients with ventilated hospital-acquired bacterial pneumonia/ventilator-associated bacterial pneumonia from a health care payer perspective.

**Methods:**

A hybrid decision-tree Markov decision-analytic model with a 5-year time horizon were developed to estimate costs and quality-adjusted life-years and to calculate the incremental cost-effectiveness ratio associated with ceftolozane/tazobactam and meropenem in the treatment of patients with ventilated hospital-acquired bacterial pneumonia/ventilator-associated bacterial pneumonia. Clinical outcomes were based on the ASPECT-NP study, costs were based on the national fee schedule of 2022, and utilities were based on published data. One-way sensitivity analysis and probabilistic sensitivity analysis were also conducted to assess the robustness of our modeled estimates.

**Results:**

According to our base-case analysis, compared with meropenem, ceftolozane/tazobactam increased the total costs by 424,731.22 yen (£2,626.96) and increased the quality-adjusted life-years by 0.17, resulting in an incremental cost-effectiveness ratio of 2,548,738 yen (£15,763.94) per quality-adjusted life-year gained for ceftolozane/tazobactam compared with meropenem. One-way sensitivity analysis showed that although the incremental cost-effectiveness ratio remained below 5,000,000 yen (£30,925) for most of the parameters, the incremental net monetary benefit may have been less than 0 depending on the treatment efficacy outcome, especially the cure rate and mortality rate for MEPM and mortality rate for CTZ/TAZ. 53.4% of the PSA simulations demonstrated that CTZ/TAZ was more cost-effective than MEPM was.

**Conclusion:**

Although incremental cost-effectiveness ratio was below ￥5,000,000 in base-case analysis, whether ceftolozane/tazobactam is a cost-effective alternative to meropenem for ventilated hospital-acquired bacterial pneumonia/ventilator-associated bacterial pneumonia in Japan remains uncertain. Future research should examine the unobserved heterogeneity across patient subgroups and decision-making settings, to characterise decision uncertainty and its consequences so as to assess whether additional research is required.

**Supplementary Information:**

The online version contains supplementary material available at 10.1186/s12913-024-10883-7.

## Introduction

### Background Information

Health care-associated infection (HAI) is an infection caused in health care facilities, and many cases are caused by antibiotic-resistant bacteria [1]. HAI has high impacts on morbidity, mortality, and economic burden. In particular, antibiotic-resistant gram-negative bacteria, such as 3rd generation cephalosporin-resistant (3GCR) *Enterobacterales*, carbapenem-resistant *Enterobacterales* (CRE), and carbapenem-resistant *Pseudomonas aeruginosa,* are high threats. These are the priority pathogens that require regulatory and development actions due to their high mortality rates and significant threat to public health [1].

The emergence of antibiotic-resistant bacteria has been a serious problem worldwide [2, 3]. In Japan, the prevalence of 3GCR gram-negative bacteria has been increasing annually; in 2017, the resistance rate was 17.4% for *E. coli* and 6.1% for *K. pneumoniae*. There were 2,333 patients with CRE infection in 2019, and this number has been increasing since 2014. These values are lower than those in Europe and the United States but are likely to increase further in the future [4].

Resistant bacterial infections are associated with high mortality. One meta-analysis showed that patients with 3GCR *E. coli* infections had significantly greater odds of 30-day mortality (sOR 2.02, 95% CI [1.66–2.46], *p* < 0.001) and all-cause mortality (sOR 2.27, 95% CI [1.92–2.70], *p* < 0.001) than patients with susceptible bacterial infections did, based on random effect meta-analysis [5]. Another meta-analysis also showed that the association between CRE infection and mortality rate was 2.85 (adjusted OR, 95% CI [1.88–4.30]) [6].

Additionally, economic and patient burdens are problematic. A report from the Centers for Disease Control and Prevention (CDC) estimated that in 2017, the attributable health care costs were 1.2 billion dollars (973 million pounds, from the annual average exchange rates in 2022) for 3GCR gram-negative bacterial infection and 130 million dollars (105 million pounds) for CRE infection in the United States [7]. This difference was mainly influenced by the length of stay (LOS). One retrospective cohort study reported that, compared with susceptible infection, 3GCR gram-negative bacterial infection was associated with 1.58 (95% CI; [0.84–2.31]) more days of stay [8]. This study additionally reported that the additional treatment cost per patient was 420 pounds, 366,600 pounds per year. Another study reported that the cost of a single CRE infection was $29,157 (£23,646) from hospital charges for additional length of stay, $15,647 (£12,690) from third-party payers for hospitalization, drug treatment, etc., and $58,692-$86,940 (£47,599-£70,508) from social perspectives for production losses [9].

In Japan, treatment of severe resistant bacterial infections has centered on carbapenems, and carbapenem is the last resort for the treatment of bacterial infections [10]. However, the emergence of carbapenem-resistant bacteria due to the increased use of carbapenems has been a concern. The recommended treatment approach is to de-escalate to narrow-range antimicrobial agents that can cover the causative organisms on the basis of susceptibility results. By preventing the overuse of carbapenems, the emergence of resistant pathogens can be suppressed.

As a new treatment option, ceftolozane/tazobactam (CTZ/TAZ) was approved in Japan in 2019. This drug combines ceftolozane, a beta-lactam, with tazobactam, a beta-lactamase inhibitor, to treat infections caused by beta-lactamase-resistant bacteria [11] and has attracted increased amounts of attention as a means of preserving carbapenems.

All phase III clinical studies for complicated urinary tract infection (cUTI), complicated intra-abdominal infection (cIAI), and ventilated hospital-acquired bacterial infection/ventilator-associated bacterial infection (vHABP/VABP) demonstrated that CTZ/TAZ was noninferior to and equally safe as the standard of care [12–14]. Additionally, an in vitro study showed that the development of CTZ/TAZ resistance in *P. aeruginosa* was much slower than that of ceftazidime, meropenem, and ciprofloxacin [15]. *P. aeruginosa* developed resistance to meropenem, ceftazidime, and ciprofloxacin within 4 to 6 days, but did not develop resistance to CTZ/TAZ after 14 days.

In a clinical guideline, the use of CTZ/TAZ was recommended for patients with severe infections or when there was difficulty treating multidrug-resistant *P. aeruginosa* [16, 17].

One paper from the United States [18] found CTZ/TAZ to be cost effective for vHABP/VABP; however, there has been no CEA in a Japanese clinical setting. Some of the values from the U.S. paper were applied in this paper, but given the differences between Japan and the U.S. in terms of medical insurance system, medical cost, and willingness to pay thresholds, new results might emerge when assessing cost-effectiveness in Japan.

### Objectives

The objective of this study was to assess the cost-effectiveness of CTZ/TAZ compared with MEPM for patients with severe resistant bacterial infections, especially vHABP and VABP, since it is one of the most common HAIs and a major cause of death among Japanese people.

The hypothesis of this study was that CTZ/TAZ would be a more cost-effective treatment than MEPM for patients with vHABP/VABP. We believe that the present results will provide one consideration when choosing antibiotics by examining whether CTZ/TAZ is not only an effective treatment but also a desirable treatment option economically.

## Methods

### Model population

The study population consisted of the microbiological intention-to-treat (mITT) population in the ASPECT-NP study [14], which consisted of adult patients (18 years or older) with vHABP/VABP who were admitted to the intensive care unit (ICU) and had at least one gram-negative bacterial respiratory pathogen isolated from the baseline culture that was susceptible to CTZ/TAZ and MEPM. We considered the mITT population to be more relevant than the intention-to-treat population because in actual medical settings, CTZ/TAZ is used after susceptibility testing results are confirmed with gram-negative bacteria.

### Model structure

**Fig. 1:**
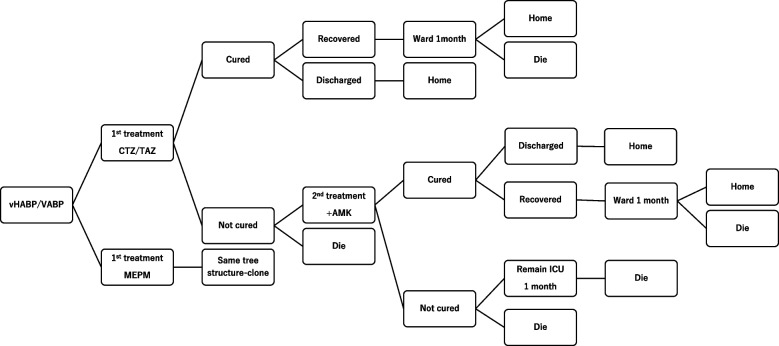
Patient flow chart

Based on a previously published US model and other studies [18–20], a hybrid decision-tree Markov decision-analytic model were developed in TreeAge Pro Health care 2022 (TreeAge Software, Inc., Williamstown, MA) for the treatment pathway in which vHABP/VABP patients receive CTZ/TAZ or MEPM (Fig. 1). The MEPM was chosen as a comparator since it is a standard treatment option according to Japanese guidelines and was used as a comparator in the ASPECT-NP study [10].

The decision tree focused on antibacterial therapy and patient response. Patient response was classified as “cured” or “not cured”. Patients entering the model were first treated with 2 g of CTZ, 1 g of TAZ or 1 g of MEPM every 8 h for 8–14 days. Cured patients were discharged or transferred to the general ward. Based on the results of the ASPECT-NP study, 50.6% of the cured patients in the CTZTAZ group and 56.7% in the MEPM group were discharged. Patients transferred to the general ward were assumed to be discharged afterward. Patients who were not cured either died or were removed from second-line treatment. In the second-line treatment, patients were treated with 200 mg of amikacin (AMK) every 12 h for 7 days in addition to the drugs used in the first-line treatment. Cured patients were discharged or transferred to a general ward as in the first-line treatment. Patients who were not cured were assumed to have died or to be continuously admitted to the ICU. These settings were based on the substudy of the ASPECT-NP study [21].

A 5-year time horizon was adopted. This finding was consistent with those of other similar studies [19, 20]. The analysis was conducted from the health care payer perspective and considered direct medical costs only; costs and quality-adjusted life-years (QALYs) were discounted at an annual rate of 2.0%, according to the Japanese guidelines [22].

### Model inputs and data sources

#### Clinical inputs

The key clinical input parameters are listed in Table 1.

**Table 1:** Key clinical inputs

Efficacy (%)	Value	Lower	Upper	Source
Clinical cure (1^st^) of CTZ/TAZ	60.7	61.0	72.0	[14, 21]
Clinical cure (1^st^) of MEPM	57.1	49.0	75.0	
Mortality (1^st^) of CTZ/TAZ	20.1	20.0	31.0	
Mortality (1^st^) of MEPM	25.5	12.0	28.0	
Clinical cure (2^nd^) of CTZ/TAZ	56.8	57.1	69.2	
Clinical cure (2^nd^) of MEPM	52.8	43.9	72.5	
Mortality (2^nd^) of CTZ/TAZ	24.1	24.0	37.2	
Mortality (2^nd^) of MEPM	30.6	14.4	33.6	
**Administration duration (Day)**				
1^st^-line treatment of CTZ/TAZ	8	7.64	8.36	[18]
1^st^-line treatment of MEPM	8.23	7.86	8.6	
2^nd^-line treatment of CTZ/TAZ	7	4	10	[10]
2^nd^-line treatment of MEPM	7	4	10	
**ICU LOS (day)**				
1^st^-line treatment of CTZ/TAZ	28.0	16	28	[21]
1^st^-line treatment of MEPM	24.0	15	28	
2^nd^-line treatment of CTZ/TAZ	21.0	20	23	
2^nd^-line treatment of MEPM	21.0	20	23	
**Mechanical ventilation duration (Day)**				
1^st^-line treatment of CTZ/TAZ	12	5	28	[21]
1^st^-line treatment of MEPM	13	6	28	
2^nd^-line treatment of CTZ/TAZ	12	5	28	
2^nd^-line treatment of MEPM	12	5	28	
**Adverse Event rate (%)**				
Septic shock of CTZ/TAZ	3.6			[14]
Multiorgan failure of CTZ/TAZ	3.9			
Acute cardiac failure of CTZ/TAZ	2.5			
Septic shock of MEPM	4.7			
Multiorgan failure of MEPM	2.8			
Acute cardiac failure of MEPM	2.2			
Acute renal failure of AMK	6.0	0.0	27.6	[23]

*CTZ/TAZ* Ceftolozane/Tazobactam, *MEPM* Meropenem, *AMK* Amikacin, *LOS* length of stay, *ICU* Intensive Care Unit

##### Efficacy

Clinical efficacy and all-cause mortality rates for CTZ/TAZ and MEPM were sourced from the ASPECT-NP study. For second-line treatment, patients were treated with first-line treatment plus AMK, based on the JAID/JSC Guide to Clinical Management of Infectious Diseases 2019 [10] and an expert opinion. Since there were no publications on the efficacy of AMK when administered in combination with first-line treatment, the efficacy of the second-line treatment was assumed to be equivalent to that of the first-line treatment. To take into account the additional burden of failure in the first-line treatment, the clinical efficacy of the second-line treatment would be reduced by 10%, and the mortality rate would be increased by 20% on the basis of other studies [19, 24]. The mortality rate for patients who were cured by the treatment and transferred from the ICU to a general ward was set at 7.6% in accordance with the literature [25]. When people were discharged home, it was assumed that they died at Japan’s natural mortality rate. The age-dependent mortality rate was obtained from the life table reported by the Ministry of Health, Labour, and Welfare in Japan [26].

##### ICU length of stay and long-term treatment

The length of stay (LOS) in the ICU for first-line treatment was 28 days and 24 days for the CTZ/TAZ and MEPM groups, respectively, from the ASPECT-NP study [21]. Patients cured by this treatment were discharged after treatment was completed, and those who recovered and were transferred to the general ward were assumed to be discharged after one month. Patients who moved to the second-line treatment were assumed to have an additional 21 days of ICU admission for the second-line treatment. Patients who were not cured by second-line treatment were assumed to die after one month. These criteria were established based on the literature [27, 28].

##### Adverse events

AEs associated with CTZ/TAZ and MEPM were sourced from the ASPECT-NP study and included 2–3 AEs, which are considered fatal, expensive to treat, and associated with a higher incidence rate. AEs associated with AMK were sourced from the literature [23].

#### Utility inputs

Given that utility values were not obtained from the ASPECT-NP study, the utility of pneumonia patients who were on a ventilator and admitted to the ICU was sourced from a literature search. These values are listed in Table 2. The disutility of AEs was omitted because the disutility was very small and the frequency of AEs was low, and consequently, the effect on the results was very small.

**Table 2:** Key cost and utility inputs

**Cost (Yen)**	**Frequency**	**Value**	**Lower**	**Upper**	**Source**
**Drug cost**					
CTZ/TAZ	Daily	36,414			[29]
MEPM	Daily	2,589			
AMK	Daily	714			
**Hospital resource**					
Mechanical ventilation	Daily (-Day 14)	9,500			[30]
	Daily (Day 15-)	8,150			
**Adverse event cost**					
Septic shock	Per event	504,885		740,757	[10, 14, 29–33]
Multiorgan failure	Per event	504,885		740,757	
Acute cardiac failure	Per event	182,758		189,860	
Acute renal failure	Per event	94,800			
**Utility**	**Value**		**Lower**	**Upper**	**Source**
Hospitalization with ventilator in ICU	-0.39		-0.402	0.3	[34]
Cured and discharged	0.8		0.66	1.0	
Recovered in general ward	0.77		0.43	0.82	[35, 36]

*CTZ/TAZ* ceftolozane/tazobactam, *MEPM* Meropenem, *AMK* Amikacin, *ICU* Intensive Care Unit

#### Cost inputs

Drug costs were calculated using the ASPECT-NP study data, dosing regimens, and Japanese medical service fees applied in 2022 [30]. The same duration of drug administration was applied to both cured and not cured patients. Hospitalization costs were sourced from the national fee schedule. The cost of treating AEs was established based on established guidelines (The JAID/JSC Guide to Clinical Management of Infectious Diseases 2019, The Japanese Guidelines for the Management of Sepsis 2012, and Guidelines for Diagnosis and Treatment of Acute and Chronic Heart Failure 2017) and expert opinion on the treatment details [10, 31, 32], and the treatment cost was calculated based on the national fee schedule, frequency of AEs in the ASPECT-NP study and the literature. These are listed in Table 2.

### Analysis

The model compared the cost-effectiveness of CTZ/TAZ versus MEPM from a health care payer perspective, considering only direct medical costs.

The primary outcome was the incremental cost per QALY gained. The outcomes included clinical outcome, total health care cost, and QALYs.

A willingness-to-pay threshold of 5,000,000 yen (£30,925) per QALY was used to implement the incremental cost-effectiveness ratio (ICER) according to the cost-effectiveness evaluation scheme in Japan.

One-way sensitivity analysis (OWSA) was conducted to test the uncertainty of the model input parameters by varying the model input parameters. We conducted a literature review and identified the maximum and minimum parameters. For the clinical cure rate and mortality rate, we conducted a meta-analysis, calculated the 95% CIs and used them as the maximum and minimum parameters. The discount rate was analyzed in the range of 0–4%.

Probabilistic sensitivity analysis (PSA) assigned distributions to each parameter and ran 1,000 simulations to assess the robustness of our modeled estimates. The parameters and distributions are listed in Appendix 1.

## Results

### Base case results

The base case results are shown in Table 3. Over a 5-year time horizon, the total incremental costs of CTZ/TAZ were 424,731.22 Yen (£2,626) and 2,955,300.18 Yen (£18,278) for CTZ/TAZ vs. 2,530,568.97 Yen (£15,651) for MEPM, and the incremental QALYs were 0.17 and 2.35 for CTZ/TAZ vs. 2.18 for MEPM. The ICER for CTZ/TAZ compared to MEPM was 2,548,738 Yen (£15,763) per QALY gained. The corresponding ICER of 2,548,738 Yen (£15,763) per QALY gained is substantially below the threshold of 5,000,000 Yen (£30,925), indicating that CTZ/TAZ may be considered a cost-effective treatment option.

**Table 3:** Base case results

Treatment	Total		Incremental		ICER (Cost/QALY)
	**Costs (Yen)**	**QALYs**	**Costs (Yen)**	**QALYs**	
MEPM	2,530,568.97	2.18			
CTZ/TAZ	2,955,300.18	2.35	424,731.22	0.17	2,548,738

*CTZ/TAZ* ceftolozane/tazobactam, *MEPM* Meropenem, *QALYs* Quality adjusted life years, *ICER* Incremental cost-effectiveness ratio

The results revealed incremental benefits of CTZ/TAZ compared with MEPM in terms of an increase in the cure rate; 60.6% vs. 57.1% for the first-line treatment; 56.8% vs. 52.8% for the second-line treatment; and a decrease in the mortality rate—20.5% vs. 25.5% for the first-line treatment and 24.1% vs. 30.6% for the second-line treatment (Table 4). The breakdown cost results showed that the drug costs were as follows: 291,312 yen (£1,801) vs. 21,307.4 yen 7 (£131) for the first-line treatment and 259,896 yen (£1,607) vs. 23,121 yen (£143) for the second-line treatment. Although CTZ/TAZ is more expensive than MEPM, the greater cure rate saves hospitalization and resource costs, partially offsetting the cost difference.

**Table 4:** Breakdown of the base-case results

	**CTZ/TAZ**	**MEPM**
**Clinical outcomes (%)**		
Cure rate (1^st^)	60.6	57.1
Cure rate (2^nd^)	56.8	52.8
Mortality rate (1^st)^	20.1	25.5
Mortality rate (2^nd^)	24.1	30.6
AE rate (Septic shock)	3.6	4.7
AE rate (Multiorgan failure)	3.9	2.8
AE rate (Cardiac failure acute)	2.5	2.2
AMK related AE rate (Acute renal failure)	6	6
**QALYs**	2.35	2.18
**Cost estimates (Yen)**		
Drug (1^st^)	291,312	21,307.47
Drug (2^nd^)	259,896	23,121
Hospitalization (1^st^)	2,136,960	2,063,280
Hospitalization (2^nd^)	350,340	358,020
Mechanical ventilation (1^st^)	114,000	123,500
Mechanical ventilation (2^nd^)	97,800	97,800
AE treatment (1^st^)	42,435.33	41,887.05
AE treatment (2^nd^)	48,123.33	47,575.05
Long-term care in ICU	495,000	495,000
Long-term care in ward	495,000	495,000

*CTZ/TAZ* ceftolozane/tazobactam, *MEPM* Meropenem, *AMK* Amikacin, *AE* Adverse event, *QALYs* Quality adjusted life years, *ICU* Intensive Care Unit

For QALYs, CTZ/TAZ had a gain of 0.17 QALYs over 5 years, with a higher cure rate and lower mortality than MEPM.

### OWSA results

**Fig. 2:**
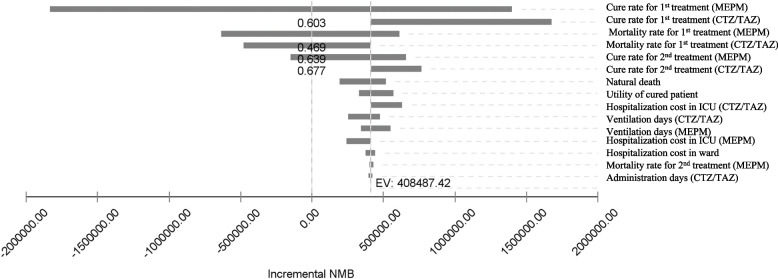
OWSA (Tornado diagram) for CTZ/TAZ versus MEPM CTZ/TAZ = ceftolozane/tazobactam, MEPM = Meropenem, ICU = Intensive Care Unit, AE = Adverse Events

OWSA was used to test uncertainty in the base-case ICER based on the upper and lower bounds of model inputs in a tornado diagram. Figure 2 shows the change in the incremental net monetary benefit (INMB) across the 15 most influential parameters. The INMB results were most influential on the cure rate of MEPM when applied as a first-line treatment. The results also showed that mortality rate and hospital resource costs had a relative impact on INMB. It was found that the INMB may be less than 0 depending on the treatment efficacy outcome, especially the cure rate and mortality rate for MEPM and mortality rate for CTZ/TAZ.

### PSA results

**Fig. 3:**
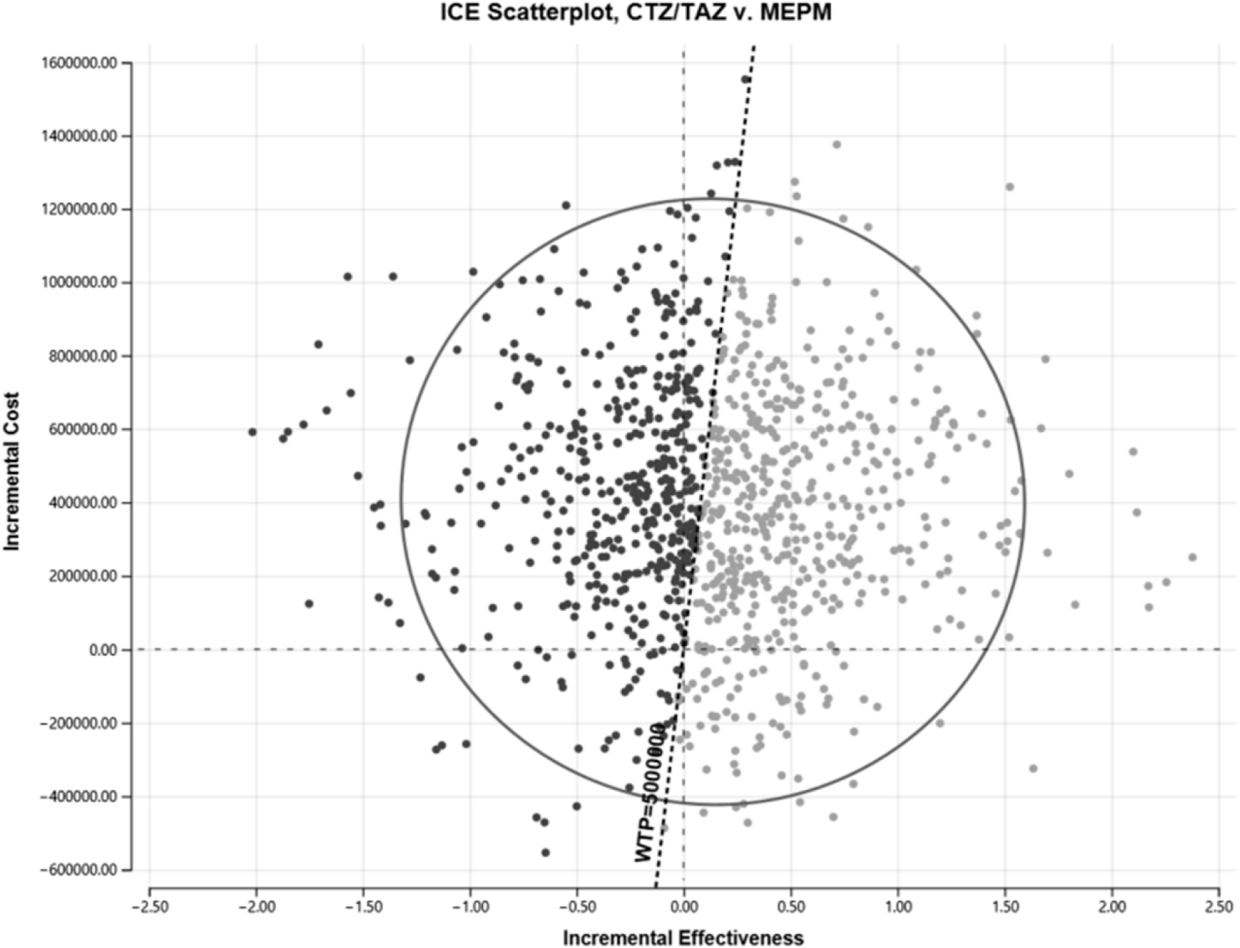
Probabilistic sensitivity analysis for CTZ/TAZ versus MEPM CTZ/TAZ = ceftolozane/tazobactam, MEPM = Meropenem

**Fig. 4:**
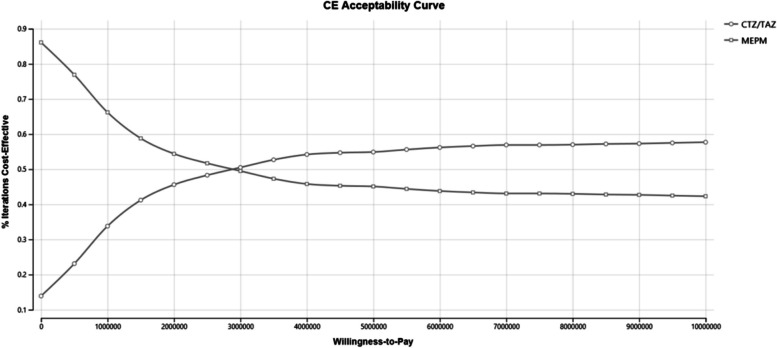
Acceptability curve for CTZ/TAZ versus MEPM CTZ/TAZ = ceftolozane/tazobactam, MEPM = Meropenem

For the PSA, the incremental cost and incremental effectiveness at the simulation points are plotted in Fig. 3. The PSA results revealed that more than half (53.4%) of the plots were located in the area where the ICER was less than 5,000,000 yen (£30,925) (Table 5). The cost-effectiveness acceptability curves for PSA showed that within the WTP of 5,000,000 yen (£30,925), CTZ/TAZ is considered more likely to be cost effective (Fig. 4). However, since the probability is almost half, it is controversial whether CTZ/TAZ is a cost-effective treatment. Additionally, how increasing the WTP threshold from £30,925 would not significantly change the probability of the new intervention being costeffective, remaining below 60% with values three times higher.

**Table 5:** Probability of PSA for CTZ/TAZ versus MEPM

Strategy	Optimal (%)
CTZ/TAZ	53.3
MEPM	46.7

*CTZ/TAZ* ceftolozane/tazobactam, *MEPM* Meropenem, *PSA* Probabilistic sensitivity analysis

## Discussion

The emergence of resistant bacteria has led to an increased reliance on carbapenems for severe infections, and this reliance has led to increased CRE. CTZ/TAZ was recently approved in Japan for the treatment of severe infections caused by β-lactamase-producing resistant bacteria and is one way to prevent increased use of carbapenems. Although CTZ/TAZ is more expensive than MEPM, the selection of a therapeutic option should take into account the efficacy of the drug and the cost of hospitalization and resource use.

In this study, a model was developed in which CTZ/TAZ or MEPM was used as the first-line treatment and AMK was added as a second-line treatment.

The results showed that the incremental QALYs were 0.17 and that the incremental costs were 424,731.22 yen (£2,626), resulting in an ICER of 2,548,738 yen (£15,763) per QALY gained compared with that of the MEPM, which was well below the threshold of 5,000,000 yen (£30,925) per QALY. In addition, the OWSA resulted in an ICER below 5,000,000 yen (£30,925) for most of the parameters. We found that the INMB is less than 0 when the cure rate of MEPM is above a certain value, when the mortality rate of MEPM is less than a certain value, or when the mortality rate of CTZ/TAZ is less than a certain value. Therefore, CTZ/TAZ is considered to be a treatment option when MEPM is not expected to be effective. More than half (53.4%) of the PSA simulations revealed that CTZ/TAZ was more cost-effective than MEPM was. However, this result also means that there is a 46% probability that the MEPM is cost-effective, and Fig. 4 indicates that the probability of cost-effectiveness of CTZ/TAZ does not increase significantly as the willingness-to-pay threshold increases. Therefore, the PSA test result indicates that, in some cases, CTZ/TAZ may not be a cost-effective option.

The ASPECT-NP study showed that CTZ/TAZ has greater efficacy than does the MEPM, and the ICER of the base case was less than the WTP; thus, CTZ/TAZ can be a treatment option for vHABP/VABP in Japan.

This is the first study to evaluate the CEA level in the CTZ/TAZ region for vHABP/VABP in Japan. Previously published papers in the US have shown CTZ/TAZ to be cost effective. In our study, we followed the design of several previous studies and made some modifications. Patient flow was developed based on previous studies. Model inputs were obtained from the ASPECT-NP study [14], clinical trials with similar patient characteristics, observational studies, similar CEA articles, expert opinions, and local databases. Some assumptions were made regarding the lack of data. These approaches were similar to those used in other CEA studies.

In contrast, the model in this study was modified to be more similar to that used in actual clinical practice. We included the flow of patients after completing antimicrobial therapy. In addition, we used a different time horizon from that used in the previous US study since a 5-year time horizon was sufficient to cover the episode of the infection and its long-term impact. Moreover, the baseline utility for vHABP/VABP patients was modified. Although the utility for patients with MRSA during and after ICU admission was examined in a previous US study [18], in this study, utility values were sourced from articles that defined pneumonia patients treated with a ventilator in the ICU. For cost inputs, since this is the CEA from the health care payer perspective, all costs were calculated using the national fee schedule in 2022 [29, 30].

Even in light of the modifications described above, this study revealed that CTZ/TAZ was cost effective.

### Limitations

This study has several limitations. First, the patient demographic, safety, and efficacy data were taken directly from the ASPECT-NP study population, which included severely ill patients who used mechanical ventilation and had at least one gram-negative pathogen; therefore, the results may not be generalizable to HABP/VABP patients overall. Additionally, the use of bacterial pathogens may not be generalizable to overall HABP/VABP patients in Japan. However, since the data from the ASPECT-NP study showed no domestic or international differences in the health care environment or in the susceptibility of each causative pathogen to CTZ/TAZ and no ethnic differences in PK between Japanese and non-Japanese individuals, we believe that the results of this study are at least applicable to Japanese patients with HABP/VABP caused by gram-negative pathogens [37].

Second, several assumptions were applied in this study due to the lack of data. Specifically, it was assumed that patients who recovered from this treatment were transferred to the general ward and discharged after one month, and patients who could not be cured during secondary treatment died after one month. This approach is based on the literature. In addition, efficacy data for the second-line treatment were also obtained from similar CEA reports, as no data were available. In fact, second-line treatment may not be first-line treatment + AMK but may be selected based on the susceptibility testing results. However, for the purpose of setting up the model, we chose AMK. The 2019 JAID/JSC Guide to Clinical Management of Infectious Diseases also states that AMK is the second-line treatment for multidrug-resistant bacterial infection, and this recommendation has been endorsed by an expert. In addition, if a second-line drug is selected based on the sensitivity testing results, a higher efficacy is expected, and the ICER can be decreased because of the lower cost of treatment. Therefore, this analysis is considered conservative, and the conclusion is that CTZ/TAZ is still cost effective.

Third, to keep the model simple, a few factors were not included in the model. The rate of susceptibility to CTZ/TAZ was not specifically considered in the model. However, the susceptibility rate is as high as 87–98% in Japan [38, 39], so we believe that this difference would not have a significant impact on the results. In addition, this study did not consider the emergence of pathogens resistant to CTZ/TAZ. However, the impact of resistant pathogens on the cost-effectiveness of CTZ/TAZ would be minimal since CTZ/TAZ resistance development is slow or very limited according to an in vitro study [15].

Fourth, in the ASPECT-NP study, the dosage of MEPM was 1 g three times daily as a 1-h infusion, but this dosage might be low for severe infections caused by resistant bacteria, and the infusion time might have been too short. Although the maximum daily dose in the MEPM package insert is 3 g, the JAID/JSC guide [10] recommends 1–2 g 3 times daily for multidrug-resistant organisms. Moreover, a longer infusion time, such as 3 h, is recommended for severe cases [40, 41]. If the MEPM dosage had been set at 2 g received three times a day via 3 h of infusion, the efficacy of MEPM would have been higher, and the ICER would have been different.

There are two factors that should be considered in future research. First, disease transmission should be considered if infections can be treated early and if, in the early stages, the risk of transmission of infectious diseases can be reduced, which will have a positive impact on clinical settings. Second, because the comparator was MEPM, we did not include patients with HABP/VABP due to carbapenem-resistant bacteria; however, since CTZ/TAZ is also effective against some of these bacteria, patients with vHABP/VABP carbapenem-resistant bacteria should also be included. These factors can be included in the model for future study.

### Implications for practice

The choice of antibiotics for vHABP/VABP patients should be determined by considering various factors, such as patient medical history, local resistance data, and susceptibility testing results; however, cost-effectiveness considerations should also be taken into account.

For severe resistant bacterial infections, carbapenems tend to be used due to the limited choice of available antibiotics, costs, and the wide spectrum. However, based on the results of this study, CTZ/TAZ can be considered a good treatment option for vHABP/VABP patients, for whom gram-negative bacteria are likely to be causative pathogens. However, CTZ/TAZ may not always be a cost-effective option, and the treatment option should be chosen based on a combination of the patient's history and test results for causative organisms.

## Conclusions

The results of base case analysis indicated that CTZ/TAZ is a cost-effective alternative to MEPM in vHABP/VABP in Japan. This is because of the greater rate of clinical cure for CTZ/TAZ than for MEPM, which led to a reduction in hospital resources.

The findings of this study support the use of CTZ/TAZ as an alternative treatment option for vHABP/VABP patients with a likelihood of gram-negative bacteria as the causative pathogens. It should be noted, however, that the results of this study are controversial, since the probability of CTZ/TAZ being a cost-effective treatment is almost half according to the PSA results.

### Supplementary Information

**Supplementary Material 1.**

## Data Availability

The dataset supporting the conclusions of this article is included within the Appendix 1.

## Abbreviations

3GCR: 3rd generation cephalosporin resistant
AE: adverse events
AMK: amikacin
CDC: Centers for Disease Control and Prevention
CEA: cost-effectiveness analysis
cIAI: complicated intra-abdominal infection
CRE: carbapenem-resistant Enterobacterales
CTZ/TAZ: ceftolozane/tazobactam
cUTI: complicated urinary tract infection
HAI: health care-associated infections
ICU: intensive care unit
INMB: incremental net monetary benefit
LOS: length of stay
MEPM: meropenem
mITT: microbiological intention-to-treat
MRSA: methicillin-resistant *Staphylococcus aureus*
OWSA: one-way sensitivity analysis
PSA: probabilistic sensitivity analysis
QALYs: quality-adjusted life-years
VABP: ventilator-associated bacterial infection
vHABP: ventilated-hospital-acquired bacterial infection

## References

[1] Tacconelli E, Magrini N. Global priority list of antibiotic-resistant bacteria to guide research, discovery, and development of new antibiotics. 2017. https://www.aidsdatahub.org/sites/default/files/resource/who-global-priority-list-antibiotic-resistant-bacteria.pdf. Accessed 3 Dec 2022.

[2] Antimicrobial resistance in the EU/EEA (EARS-Net) - Annual epidemiological report for 2021. European Centre for Disease Prevention and Control. 2022. https://www.ecdc.europa.eu/en/publications-data/surveillance-antimicrobial-resistance-europe-2021. Accessed 3 Dec 2022.

[3] ESBL-producing Enterobacterales | HAI | CDC. 2021. https://www.cdc.gov/hai/organisms/ESBL.html. Accessed 3 Dec 2022.

[4] Notification status of carbapenem-resistant Enterobacteriaceae infections under the Infectious Diseases Act, 2019. https://www.niid.go.jp/niid/ja/cre-m/cre-idwrs/10319-cre-210423.html. Accessed 14 May 2023.

[5] MacKinnon MC, Sargeant JM, Pearl DL, Reid-Smith RJ, Carson CA, Parmley EJ (2020). Evaluation of the health and healthcare system burden due to antimicrobial-resistant Escherichia coli infections in humans: a systematic review and meta-analysis. Antimicrob Resist Infect Control..

[6] Soontaros S, Leelakanok N (2019). Association between carbapenem-resistant Enterobacteriaceae and death: a systematic review and meta-analysis. Am J Infect Control..

[7] CRE | HAI | CDC. 2021. https://www.cdc.gov/hai/organisms/cre/index.html. Accessed 3 Dec 2022.

[8] Naylor NR, Pouwels KB, Hope R, Green N, Henderson KL, Knight GM (2019). The health and cost burden of antibiotic resistant and susceptible Escherichia coli bacteraemia in the English hospital setting: a national retrospective cohort study. PLoS One..

[9] Bartsch SM, McKinnell JA, Mueller LE, Miller LG, Gohil SK, Huang SS (2017). Potential economic burden of carbapenem-resistant Enterobacteriaceae (CRE) in the United States. Clin Microbiol Infect..

[10] The JAID/JSC Guide to Clinical Management of Infectious Diseases Development Committee. The Jaid/JSC GUide to Clinical Management of Infectious Diseases 2019; 2019.

[11] Merck & Co., Inc., ZERBAXA combination for intravenous drip infusion. Package Insert. 2021. https://www.info.pmda.go.jp/go/pack/6139506D1020_1_04/. Accessed 3 Dec 2022.

[12] Wagenlehner FM, Umeh O, Steenbergen J, Yuan G, Darouiche RO (2015). Ceftolozane-tazobactam compared with levofloxacin in the treatment of complicated urinary-tract infections, including pyelonephritis: a randomised, double-blind, phase 3 trial (ASPECT-cUTI). The Lancet..

[13] Solomkin J, Hershberger E, Miller B, Popejoy M, Friedland I, Steenbergen J (2015). Ceftolozane/Tazobactam Plus Metronidazole for Complicated Intra-abdominal Infections in an Era of Multidrug Resistance: Results From a Randomized, Double-Blind, Phase 3 Trial (ASPECT-cIAI). Clin Infect Dis..

[14] Kollef MH, Nováček M, Kivistik Ü, Réa-Neto Á, Shime N, Martin-Loeches I (2019). Ceftolozane–tazobactam versus meropenem for treatment of nosocomial pneumonia (ASPECT-NP): a randomised, controlled, double-blind, phase 3, non-inferiority trial. Lancet Infect Dis..

[15] Cabot G, Bruchmann S, Mulet X, Zamorano L, Moyà B, Juan C (2014). Pseudomonas aeruginosa Ceftolozane-Tazobactam Resistance Development Requires Multiple Mutations Leading to Overexpression and Structural Modification of AmpC. Antimicrob Agents Chemother..

[16] IDSA. IDSA guidance on the treatment of antimicrobial-resistant gram-negative infections; 2022. https://www.idsociety.org/practice-guideline/amr-guidance/. Accessed 3 Dec 2022.

[17] Paul M, Carrara E, Retamar P, Tängdén T, Bitterman R, Bonomo RA (2022). European Society of Clinical Microbiology and Infectious Diseases (ESCMID) guidelines for the treatment of infections caused by multidrug-resistant Gram-negative bacilli (endorsed by European society of intensive care medicine). Clin Microbiol Infect..

[18] Naik J, Puzniak L, Critchlow S, Elsea D, Dillon RJ, Yang J (2021). Cost effectiveness of ceftolozane/tazobactam compared with meropenem for the treatment of patients with ventilated hospital-acquired bacterial pneumonia and ventilator-associated bacterial pneumonia. Infect Dis Ther..

[19] Tichy E, Torres A, Bassetti M, Kongnakorn T, Di Virgilio R, Irani P (2020). Cost-effectiveness comparison of ceftazidime/avibactam versus meropenem in the empirical treatment of hospital-acquired pneumonia, including ventilator-associated pneumonia in Italy. Clin Ther..

[20] Simon MS, Sfeir MM, Calfee DP, Satlin MJ (2019). Cost-effectiveness of ceftazidime-avibactam for treatment of carbapenem-resistant *enterobacteriaceae* bacteremia and pneumonia. Antimicrob Agents Chemother..

[21] Lodise T, Yang J, Puzniak LA, Dillon R, Kollef M (2020). Healthcare resource utilization of ceftolozane/tazobactam versus meropenem for ventilated nosocomial pneumonia from the randomized, controlled, double-blind ASPECT-NP trial. Infect Dis Ther.

[22] Center for Outcomes Research and Economic Evaluation for Health. Guidelines for Analysis of Cost-Effectiveness Evaluation in the Central Social Insurance Medical Council, 2nd ed; 2019. https://c2h.niph.go.jp/tools/guideline/guideline_en.pdf. Accessed 3 Dec 2022.

[23] Paul M, Lador A, Grozinsky-Glasberg S, Leibovici L. Beta lactam antibiotic monotherapy versus beta lactam-aminoglycoside antibiotic combination therapy for sepsis (Review). Cochrane Database of Syst Rev. 2014(1):CD003344. 10.1002/14651858.CD003344.pub3. 10.1002/14651858.CD003344.pub3 PMC6517128 24395715

[24] Kongnakorn T, Eckmann C, Bassetti M, Tichy E, Di Virgilio R, Baillon-Plot N (2019). Cost-effectiveness analysis comparing ceftazidime/avibactam (CAZ-AVI) as empirical treatment comparing to ceftolozane/tazobactam and to meropenem for complicated intra-abdominal infection (cIAI). Antimicrob Resist Infect Control..

[25] Hosein FS, Roberts DJ, Turin TC, Zygun D, Ghali WA, Stelfox HT (2014). A meta-analysis to derive literature-based benchmarks for readmission and hospital mortality after patient discharge from intensive care. Crit Care..

[26] Ministry of Health Labour and Welfare. Simplified life table 2021. https://www.mhlw.go.jp/toukei/saikin/hw/life/life21/index.html. Accessed 2 Dec2022.

[27] Gallagher JC, Satlin MJ, Elabor A, Saraiya N, McCreary EK, Molnar E (2018). Ceftolozane-Tazobactam for the Treatment of Multidrug-Resistant Pseudomonas aeruginosa Infections: A Multicenter Study. Open Forum Infect Dis..

[28] Balandin B, Ballesteros D, Ruiz de Luna R, López-Vergara L, Pintado V, Sancho-González M (2021). Multicenter study of ceftolozane/tazobactam for treatment of Pseudomonas aeruginosa infections in critically ill patients. Int J Antimicrob Agents..

[29] Ministry of Health Labour and Welfare. Revision of medical fees for 2022. https://www.mhlw.go.jp/topics/2022/04/tp20220401-01.html. Accessed 2 Dec 2022.

[30] Ministry of Health Labour and Welfare. List of Items Listed in the NHI Drug Price Standards and Information on Generic Drugs (Effective November 16, 2022). https://www.mhlw.go.jp/topics/2022/04/tp20220401-01.html. Accessed 2 Dec 2022.

[31] Sepsis Registry Committee of the Japanese Society of Intensive Care Medicine. The Japanese Guidelines for the Management of Sepsis (2012). https://www.jsicm.org/pdf/SepsisJapan2012.pdf. Accessed 8 July 2023.

[32] Japanese Circulatory Society/Japanese Heart Failure Society. Guidelines for diagnosis and treatment of acute and chronic heart failure (2017). https://www.j-circ.or.jp/cms/wp-content/uploads/2017/06/JCS2017_tsutsui_d.pdf. Accessed 8 July 2023.

[33] Nichi-Iko Pharmaceutical Co., Ltd. Amikacin Sulfate Injection 100mg, 200mg "Nichiiko". Available at: https://s3-ap-northeast-1.amazonaws.com/medley-medicine/prescriptionpdf/530169_6123402A1184_1_02.pdf. Accessed 3 Dec 2022.

[34] Saunders R, Geogopoulos D (2018). Evaluating the cost-effectiveness of proportional-assist ventilation plus vs. pressure support ventilation in the intensive care unit in two countries. Front Public Health..

[35] Dick AW, Perencevich EN, Pogorzelska-Maziarz M, Zwanziger J, Larson EL, Stone PW (2015). A decade of investment in infection prevention: a cost-effectiveness analysis. Am J Infect Control..

[36] Andronis L, Oppong RA, Manga N, Senanayake E, Gopal S, Charman S (2018). Is the Venner-Pneux endotracheal tube system a cost-effective option for post cardiac surgery care?. Ann Thorac Surg..

[37] Merck & Co., Inc., ZERBAXA combination for intravenous drip infusion. Review report 2019. https://www.pmda.go.jp/drugs/2019/P20191217002/170050000_23100AMX00005_A100_1.pdf. Accessed 3 Dec 2022.

[38] Candel FJ, Santerre Henriksen A, Longshaw C, Yamano Y, Oliver A (2022). In vitro activity of the novel siderophore cephalosporin, cefiderocol, in Gram-negative pathogens in Europe by site of infection. Clin Microbiol Infect..

[39] Karlowsky JA, Hackel MA, Takemura M, Yamano Y, Echols R, Sahm DF (2022). In vitro susceptibility of gram-negative pathogens to Cefiderocol in five consecutive annual multinational SIDERO-WT surveillance studies, 2014 to 2019. Antimicrob Agents Chemother..

[40] Win EE, Htun KW, Tragulpiankit P, Tangtrakultham S, Montakantikul P (2022). The evaluation of Meropenem dosing regimens against Esbl-producing Escherichia coli in ICU patients using Monte Carlo simulation. Infect Drug Resist..

[41] Zhao YC, Zou Y, Xiao YW, Wang F, Zhang BK, Xiang DX (2022). Does prolonged infusion time really improve the efficacy of meropenem therapy? A prospective study in critically ill patients. Infect Dis Ther..

